# The destiny of the resistance/susceptibility against GCRV is controlled by epigenetic mechanisms in CIK cells

**DOI:** 10.1038/s41598-017-03990-5

**Published:** 2017-07-03

**Authors:** Xueying Shang, Chunrong Yang, Quanyuan Wan, Youliang Rao, Jianguo Su

**Affiliations:** 1 College of Animal Science and Technology, Northwest A&F University, Yangling, 712100 China; 2 College of Veterinary Medicine, Huazhong Agricultural University, Wuhan, 430070 China; 3 College of Fisheries, Huazhong Agricultural University, Wuhan, 430070 China; 4 Hubei Provincial Engineering Laboratory for Pond Aquaculture, Wuhan, 430070 China

## Abstract

Hemorrhagic disease caused by grass carp reovirus (GCRV) has severely threatened the grass carp (*Ctenopharyngodon idella*) cultivation industry. It is noteworthy that the resistance against GCRV infection was reported to be inheritable, and identified at both individual and cellular levels. Therefore, this work was inspired and dedicated to unravel the molecular mechanisms of fate decision post GCRV infection in related immune cells. Foremost, the resistant and susceptible CIK (*C. idella* kidney) monoclonal cells were established by single cell sorting, subculturing and infection screening successively. RNA-Seq, MeDIP-Seq and small RNA-Seq were carried out with C1 (CIK cells), R2 (resistant cells) and S3 (susceptible cells) groups. It was demonstrated that genome-wide DNA methylation, mRNA and microRNA expression levels in S3 were the highest among three groups. Transcriptome analysis elucidated that pathways associated with antioxidant activity, cell proliferation regulation, apoptosis activity and energy consuming might contribute to the decision of cell fates post infection. And a series of immune-related genes were identified differentially expressed across resistant and susceptible groups, which were negatively modulated by DNA methylation or microRNAs. To conclude, this study systematically uncovered the regulatory mechanism on the resistance from epigenetic perspective and provided potential biomarkers for future studies on resistance breeding.

## Introduction

Grass carp (*Ctenopharyngodon idella*), a member of the *Cyprinidae* family, has remarkable aquaculture value in China^1^. However, the development of the industry has been severely influenced by diversiform bacterial and viral diseases, of which grass carp hemorrhagic disease caused by grass carp reovirus (GCRV) infection is especially damaging and disturbing^2, 3^.

In a former study conducted with 10, 000 GCRV infected fish, the resistant individuals were identified within familial groups, indicating that GCRV resistance trait has high heritability^4^. Considering *C. idella* kidney (CIK) cell line stems from the major immune organ as well as exhibits significant cytopathic effect (CPE) post GCRV infection, it is customarily served as the research model for hemorrhagic disease^5^. The phenomena that a handful of survivors could always be observed post GCRV infection and further proliferate into cell clusters separately still with the resistance trait, which inspired us to reveal the regulatory mechanism on resistance against GCRV. And the discrepancies on phenotype are reflected in a profound change on gene expression, which is regulated at transcriptional and post-transcriptional levels^6^.

DNA methylation, a key epigenetic modification, generally occurs in the fifth position of cytosine (5-methylcytosine, 5-mC) and scatters over the gene body^7^. It is frequently described as a “silencing” mark acted on gene expression^6, 8^. And the transcription could be negatively modulated by aberrant methylation especially in CpG islands (CGIs) at two levels: inhibiting the binding of specific transcription factors directly or recruiting methyl-CpG-binding proteins repressing chromatin remodeling activities indirectly^9^. MicroRNAs (miRNAs), a kind of small (approximately 18–22 nucleotides), endogenous and non-coding RNA molecules, play important regulatory roles in post-transcriptional gene silencing, which are energetically involved in crucial biological processes such as proliferation, differentiation, apoptosis and development^10–12^. miRNAs could recognize the targeted mRNA sequences generally located in the 3′UTR and repress gene expression by impeding translation initiation or promoting mRNA deadenylation and degradation^13, 14^.

Accumulating evidences have demonstrated that the diversiform diseases result from aberrant DNA methylation^15, 16^. Accordingly, it could provide valuable insights into pathogenesis in cancer, mental disorders as well as several autoimmune disorders by revealing the methylation patterns of functional genes especially over genome-wide^17–20^. To date, there are an increasing number of researches revealing the regulatory mechanisms of diverse traits from DNA methylation standpoint in teleost, primarily focused on the fields of sexual development, genetic breeding and ecotype, while few studies were reported in diseases and immunology^21–24^. As the foremost regulator at post-transcriptional level, miRNAs have explicitly demonstrated the crucial roles in the development and various physiological processes in teleost fishes^25, 26^. And the landscapes of miRNAs have been composed in nine fish species involving zebrafish (*Danio rerio*), Atlantic salmon (*Salmo salar*), Fugu (*Takifugu rubripes*), Japanese medaka (*Oryzias latipes*), common carp (*Cyprinus carpio*), Tetraodon (*Tetraodon nigroviridis*), olive flounder (*Paralichthys olivaceus*), channel catfish (*Ictalurus punctatus*) and Atlantic halibut (*Hippoglossus hippoglossus*) in the latest released database (http://www.mirbase.org/)^27^. Thereafter, the miRNAs were profiled and functionally characterized in marine medaka (*Oryzias melastigma*) and nile tilapia (*Oreochromis niloticus*)^28, 29^. Recently, researchers provide comprehensive identification of miRNAs in *C. idella*, revealing the molecular responses against decabromodiphenyl ethane in natural water body and miRNA targets associated with motile *Aeromonad septicemia* ^30, 31^.

In the present study, we first established the resistant and susceptible monoclonal cell models against GCRV through rigorous screening. Further, we investigated DNA methylation over genome-wide and constructed mRNA and miRNA transcription profiling by high-throughput sequencing of the resistant and susceptible cells. By integrated analysis of DNA methylation and miRNAs with gene expression, the regulatory mechanism in *C. idella* against GCRV was revealed from the epigenetic perspective, and multiple functional genes modulated by DNA methylation and miRNAs were excavated as the potential biomarkers contributing to breeding for disease resistance (Supplementary Fig. S1). This study undoubtedly provided an original thought into pathogenesis researches on hemorrhagic disease in *C. idella*.

## Results

### Establishment of resistant and susceptible monoclonal cells

**Figure 1:**
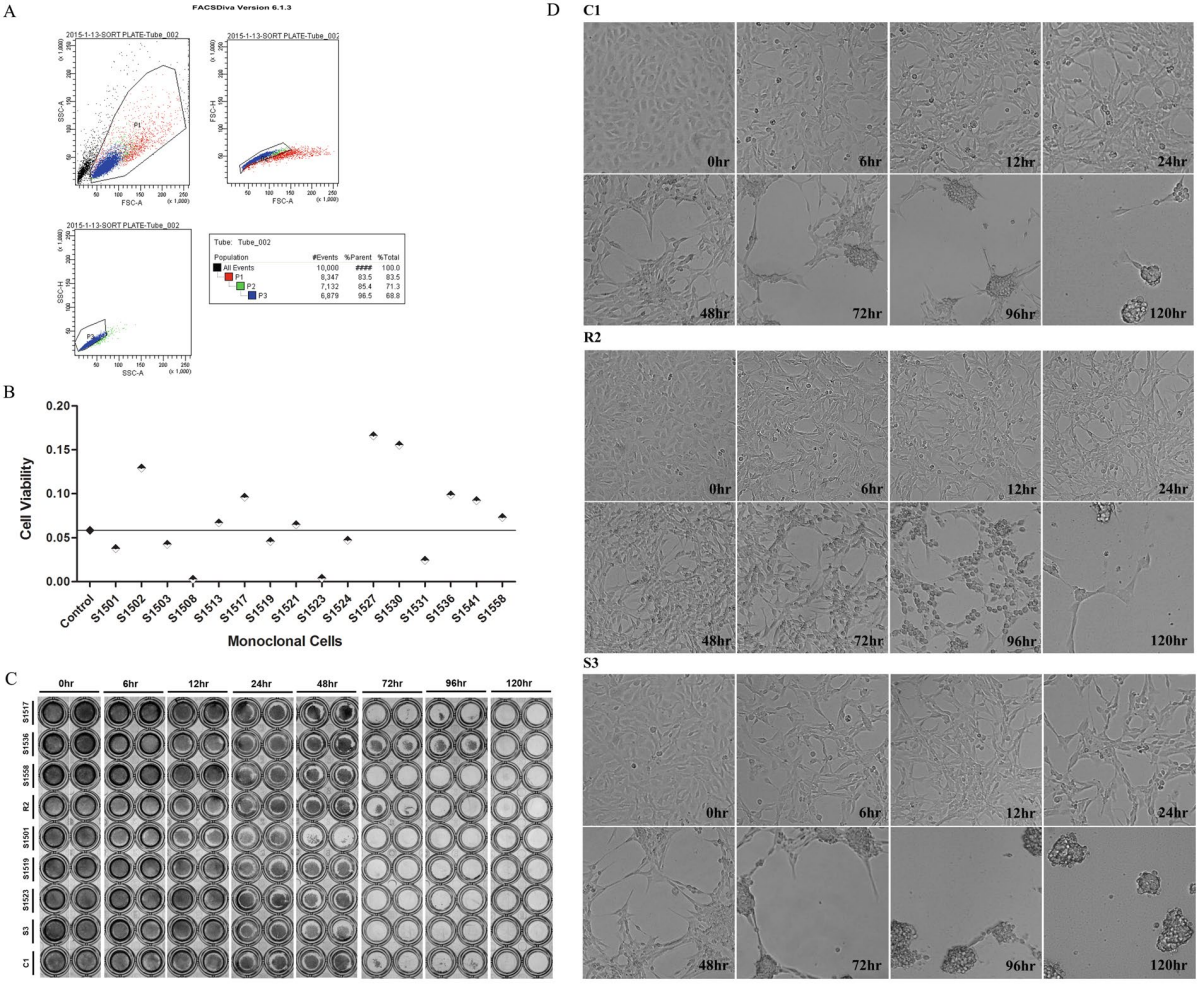
Establishment of resistant and susceptible monoclonal cells. (**A**) Single cell sorting by flow cytometry. The black spots mark dead cells in sample, the red and green spots mean adhesive cells, above cells were abandoned to ensure the vitality and purity of the sorting cells. And the remaining blue spots signify the cells used for single cell sorting. (**B**) Cell proliferation assay. The straight line signifies CV of the control CIK cells, the monoclonal cells of which CV above the line were the candidate resistant cells, while those CV under the line were preliminarily identified as susceptible cells. (**C**) Antiviral activity assay. The antiviral activities of control (C1), resistant (R2) and susceptible (S3) cells were detected at 0, 6, 12, 24, 48, 72, 96 and 120 h post GCRV infection. (**D**) CPE analysis. The morphology and CPE of C1, R2 and S3 cell groups were recorded at 0, 6, 12, 24, 48, 72, 96 and 120 h post GCRV challenge.

To establish research models for the resistant and susceptible traits, CIK cells were sorted in virtue of the flow cytometry, and 384 single cells were obtained (Fig. 1A). By subculturing, 66 monoclonal cell strains were ultimately reaped and applied for the experimental subjects screening, which were numbered S1501 to S1566 sequentially. Eight cell strains were preliminary identified as susceptible cells which exerted conspicuous CPE at 6 h post GCRV infection, mass mortality after 48 h as well as scarcely any survival 72 h later. Eight cell strains, showing no obvious CPE and still having massive living cells at 72 h post GCRV infection, were initially defined as resistant cells. By performing cell proliferation assay in combination, cell viability (CV) in S1517, S1536 and S1558 exceeded that in CIK cells, which were eventually defined as the resistant cells; and CV in S1501, S1519 and S1523 was inferior to that in control, which were ultimately confirmed as the susceptible ones. The CV situations of 16 candidate cells were shown in Fig. 1B. The morphology of the determined resistant and susceptible cell strains at 0, 6, 12, 24, 48, 72, 96 and 120 h post GCRV infection were demonstrated in Supplementary Fig. S3. To eliminate the discrepancies among cell strains, blended samples were taken as the experimental subjects embracing the resistant pool (R2) and the susceptible pool (S3), the primordial CIK cells (C1) were served as the control. The antiviral activity assay was whereafter proceeded to verify the veracity of the definition on resistant and susceptible cell strains. The results displayed that the living cell counts in the resistant cells were significantly more than those in the susceptible ones at 48 h and still existed at 96 h post GCRV infection (Fig. 1C). The cell status in C1, R2 and S3 groups at 0, 6, 12, 24, 48, 72, 96 and 120 h post GCRV infection were shown in Fig. 1D.

### mRNA expression discrepancies between R2 and S3

The high-throughput RNA sequencing (RNA-Seq) were performed to search for the discrepancies on mRNA expression profiles between the resistant and susceptible traits against GCRV. By Illumina HiSeq^TM^ 2500 paired-end (2 × 125 bp) sequencing, approximate 18.5 Gb of clean data were obtained from the 3 libraries, and 21,250 genes were identified via mapping the genome of *C. idella* ^1^. The number of clean reads eliminated the reads mapped to rRNA and the mapping ratio were listed in the Supplementary Table S5.

Abundance distribution of expression analysis demonstrated that the expression profile of S3 was in the propensity for C1 and at a higher level with respect to R2 (Supplementary Fig. S4A). In addition, C1 and S3 aggregated at one branch and were away from R2 by the clustering analysis (Supplementary Fig. S4B). Similarly, principal component analysis (PCA) (Supplementary Fig. S4C) and correlation heat map (Supplementary Fig. S4D) elucidated that S3 possessed the similar expression profile to CIK cell line. Above all, it could be demonstrated that the aboriginality of CIK cells was gravitated to the susceptible trait for the high sensitivity to GCRV.

**Figure 2:**
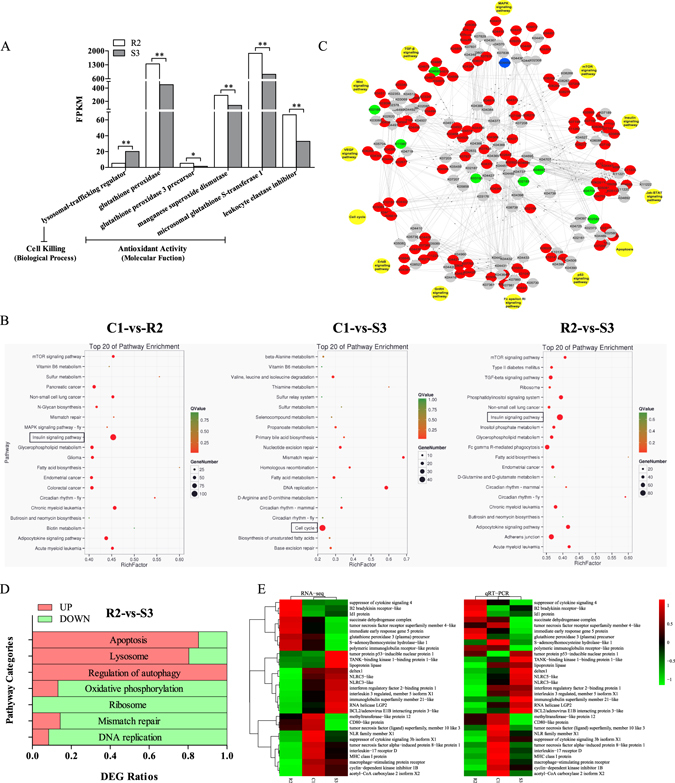
Characteristic comparisons of RNA-Seq dataset. (**A**) mRNA expression differences of six genes between R2 and S3 severally pertain to cell killing and antioxidant activity. The category names in GO analysis were labeled in brackets. (**B**) Pathway enrichment analysis of DEGs among groups. The pathways labeled by boxes indicate the one enriched the most DEGs. (**C**) The interaction network analysis of cell proliferation-related pathways. The red circles represent that all gene expressions in the corresponding in-silico knock-out (KO) analysis were higher in S3 compared to those in R2, the green signify conversely, the blue circles indicate that both up-regulated and down-regulated genes involved in the corresponding KO, and no significant DEGs are labeled by the gray circles. (**D**) The statistical analyses of DEGs enriched in seven pathways. Red signifies the ratio of higher expression genes in S3 in comparison to R2, green signifies conversely. (**E**) Validation of RNA-Seq data by qRT-PCR. The left profile shows mRNA expression patterns of 31 representative genes detected by RNA-Seq, the right one is the corresponding verification results by qRT-PCR. *EF1α* was employed as an internal control.

It was detected a mass of differentially expressed genes (DEGs) by RNA-Seq, comprising 5,889 genes between R2 and S3, 3,351 genes between C1 and S3, 6,887 genes between C1 and R2 (Supplementary Fig. S5). The percentage of up-regulated genes in S3 was more than 81.1% compared with R2, outdistancing the ratio of down-regulated ones. To demystify the molecular mechanism causing the distinct phenotypes resistance/susceptibility against GCRV, gene ontology (GO) and Kyoto encyclopedia of genes and genomes pathway database (KEGG) analyses were performed in the DEGs between R2 and S3. It was observed that the expression level of *lysosomal-trafficking regulator* was significantly higher in S3 than that in R2, which was enriched in cell killing (biological process). Conversely, five genes enriched in antioxidant activity (molecular function), including *glutathione peroxidase*, *glutathione peroxidase 3 precursor*, *manganese superoxide dismutase*, *microsomal glutathione S-transferase 1* and *leukocyte elastase inhibitor*, possessed higher expression levels in R2 than those in S3 (Fig. 2A). It could be legitimately deduced that R2 possesses more forceful antioxidant activity than S3 while S3 encounters stronger cell killing activity than R2, which might be the primary principles causing the disparate resistance/susceptibility traits against GCRV. By KEGG analysis, it was surprised to discover that DEGs between R2 and S3 were uppermost enriched in the insulin signaling pathway, likewise between C1 and R2. Additionally, DEGs between C1 and S3 mainly enriched in the cell cycle pathway (Fig. 2B). Insulin signaling pathway and cell cycle pathway were reported principally participated in cell proliferation regulation^32, 33^. Accordingly, it could be evolved that resistance and susceptibility traits discriminately existed in CIK cells might be tightly related to cell proliferation. Therefore, it was specifically essential to carry out the interaction network analysis of cell proliferation-related pathways for excavating the pivotal molecules involved in the regulation on cell proliferation (Fig. 2C). And the potential molecules were listed in the Supplementary Table S12 in detail which would lay the foundation for the future researches. Furthermore, the overwhelming majority of DEGs enriched in cell apoptosis related pathways were up-regulated in S3 compared with R2, while most DEGs enriched in cell proliferation related pathways were up-regulated in R2 rather than those in S3, which were consistent with the results yielded by GO analysis (Fig. 2D). To authenticate the reliability of RNA-Seq data as well as the rationalization of the above interpretations, mRNA expressions of 31 genes were thoroughly investigated by quantitative real-time RT-PCR (qRT-PCR). And the expression profiles of almost all genes validated by qRT-PCR were consistent with the RNA-Seq data (Fig. 2E).

### DNA methylation and small RNA profiling

**Figure 3:**
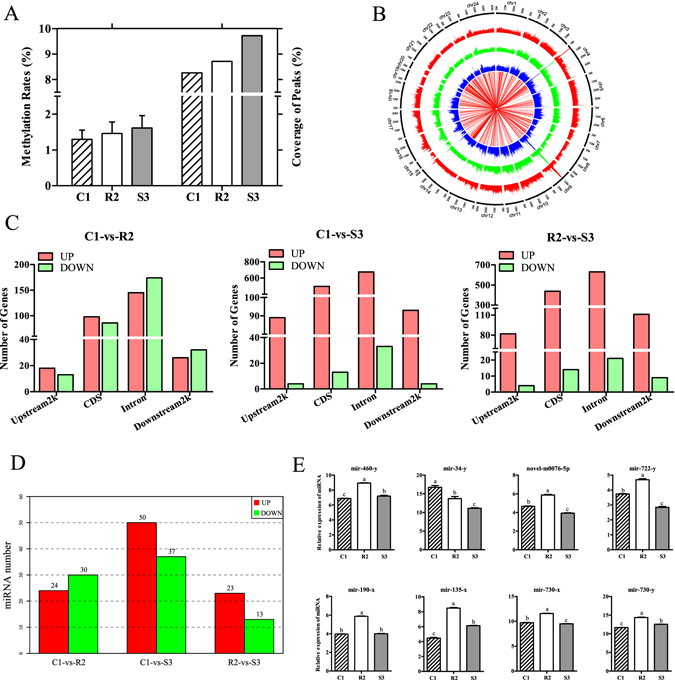
Genome-wide compound analyses of MeDIP-Seq and small RNA-Seq data. (**A**) 5-mC in total DNA. The left three columns show the methylation rates of 5-mC in C1, R2 and S3 groups detected by the MethylFlash^TM^ Methylated DNA Quantification Kit (Epigentek, USA). The right ones signify the genome coverage of peaks in C1, R2 and S3 investigated by MeDIP-Seq. (**B**) Circos plot of the DMR distribution. The blue short lines represent the DMR distribution in C1, the green ones signify that in R2, and distribution of DMR in S3 is shown by the red ones. The red/green long lines in the center indicate the comparison of DMR methylation levels between R2 and S3, in which red lines represent the DMR methylation levels are higher in S3 than those in R2, and the green ones signify on the contrary. (**C**) The statistic analyses of DMGs located in the different functional elements, including upstream2k, CDS, intron and downstream2k regions. The red columns represent the gene numbers of DMGs more in the latter than those in the former, and the green ones signify conversely. (**D**) Statistics on the number of DE-miRNAs among groups. The reds represent DE-miRNAs expressed higher in the latter than those in the former, while the greens signify on the contrary. (**E**) qRT-PCR examination of eight representative miRNAs. The abundance of miRNA was normalized to U6 small nuclear RNA.

In the present study, approximate 17.7 Gb of data bulk were obtained from three groups by MeDIP sequencing (MeDIP-Seq). The amount of the mapped reads in C1, R2 and S3 groups were 37,436,470, 37,565,439 and 45,846,648, respectively (Supplementary Table S6). Most of the methylated cytosines were in a CHH context (H = C, T, or A), and a small fraction were in CHG and CG among C1, R2 and S3 samples (Supplementary Fig. S6). Genome coverage ratio of the methylation enrichment regions addressed as peaks in S3 was 9.72% and obviously higher than coverage ratio in R2 which was just approximate 8.71% (Supplementary Table S7). It indicated that the methylation level in S3 was higher than that in R2. To verify the above statement, global DNA methylation status in three groups were detected by assay kits, and the results were essentially in agreement with the data of MeDIP-Seq (Fig. 3A). Genomic distribution of peaks showed that methylation were detected at all chromosomes. The methylation levels of numerous peaks in S3 were significantly higher than those in R2, roughly accordant with the genome coverage ratio of peaks as well as the global DNA methylation status among three groups (Fig. 3B). The distribution of peaks were subsequently analyzed in the upstream2k, coding sequence (CDS), intron and downstream2k regions. Overall, the most of peaks distributed in introns, followed by CDSs (Supplementary Fig. S7). 925 genes harbored differentially methylated regions (DMRs) on the distinct functional elements, henceforth referred to as differentially methylated genes (DMGs). The distribution of DMGs on functional elements among three samples were exhibited in Fig. 3C. To reveal the regulatory mechanisms of phenotypic differentiation from DNA methylation standpoint, DMGs between groups would be therewith made a thorough inquiry.

By high-throughput small RNA sequencing (small RNA-Seq), 1337 miRNAs were identified in three groups altogether which included 673 novel miRNAs, and the expression profiles were shown in Supplementary Fig. S8A. The majority of miRNAs were centralized at 22 nt in length tallying with the feature of animal’s miRNAs (Supplementary Fig. S8B). Chromosomal distribution of miRNAs in three groups were exhibited in Supplementary Fig. S8C. By the nucleotide bias analysis, the first nucleotide bias in all three groups was notoriously biased in favor of uracil not only in known miRNAs but in novel miRNAs (Supplementary Fig. S9A). Unexpectedly, multiple positions in both known and novel miRNAs possessed entirely discrepant nucleotide bias among three groups which might provide a meaningful perspective to dissect the regulatory mechanism of resistance/susceptibility against GCRV (Supplementary Fig. S9B). The most noteworthy was that the sequencing data unearthed 36 differentially expressed miRNAs (DE-miRNAs) between R2 and S3 groups, which were recognized as potentially major players in gene expression regulation at the post-transcriptional level (Fig. 3D). To authenticate the reliability of small RNA-Seq data, eight DE-miRNAs between R2 and S3 were randomly selected for carrying out qRT-PCR verification subsequently, displaying the coincident expression patterns compared with the sequencing results (Fig. 3E). Furthermore, DE-miRNAs binding target genes (DETs) would be implemented integrated analysis with gene transcription, aiming at bringing insights into the regulatory mechanism from the perspective of miRNAs.

### Integrated analysis of DNA methylation and gene expression

**Figure 4:**
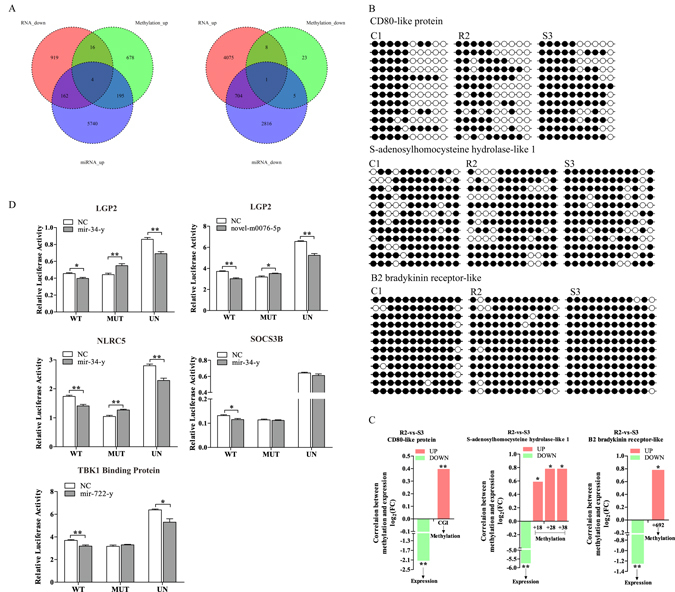
The integrated analysis and biomarker screening. (**A**) The integrated analysis of DNA methylation, miRNAs and mRNA data. The left Venn diagram reveals the regulatory mechanism that hypermethylation and overexpressed miRNAs impede mRNA expression, while the right one shows mRNA up-regulation is modulated by hypomethylation and down-regulated miRNAs. (**B**) The methylation status of candidate biomarkers were investigated by BSP. The columns show each of the CpG loci, the rows show twelve independent clones randomly selected for sequencing. The black circles signify methylation occurred in CpG loci, while the white ones label unmethylation. The statistical analysis is shown in the results section detailedly. (**C**) The correlation between DNA methylation and mRNA expression. The red columns represent log_2_(FC) of methylation levels higher in S3 than those in R2, the green ones signify log_2_(FC) of mRNA expression levels higher in R2 than those in S3. * represents the significant discrepancy (*P* < 0.05), ** represents the highly significant difference (*P* < 0.01). (**D**) Dual luciferase reporter assays for validation of miR-34-y target sites in the 3′UTR of *LGP2*, *NLRC5* and *SOCS3B*, novel-m0076-5p target site in 3′UTR of *LGP2* as well as mir-722-y target site in the 3′UTR of *TBK1 binding protein*. Luciferase assays were repeated in quadruplicate. * represents the significant discrepancy (*P* < 0.05), ** represents the extremely significant difference (*P* < 0.01).

Aberrant DNA methylation generally is associated with gene repression, particularly when it occurs at CGIs^6^. To gain insights into the regulatory mechanism of DNA methylation acted upon the phenotype of CIK cells, we carried out the integrated analysis of DMGs and negatively correlated DEGs between R2 and S3 (Fig. 4A). The results manifested that the transcription levels of 29 genes might be negatively regulated by methylation. Through in-depth analysis, three representative immune-related genes, including *CD80-like protein*, *S-adenosylhomocysteine hydrolase-like 1* (*AHCY-like 1*) and *B2 bradykinin receptor-like*, were screened out as the candidate genes for distinguishing phenotype of CIK cells, given that the methylation occurred in CGIs as the principal regulatory elements. Subsequently, bisulfite sequencing PCR (BSP) assay was performed to further corroborate the filtered results as well as excavate the specific CpG sites acted upon mRNA expressions. The methylation profiles at all CpG sites in candidate CGIs encompassed by peaks were represented in Fig. 4B. It was demonstrated that the methylation levels at total CpG sites located in the thirteenth intron’s CGI in *CD80-like protein*, +13, +28 and +38 nt in the twelfth intron’s CGI in *AHCY-like 1* as well as +692 nt in the 3′UTR’s CGI in *B2 bradykinin receptor-like* were all significantly higher in S3 than those in R2. And the statistical analysis of each CpG site in above three genes were detailedly listed in the Supplementary Tables 8–10 respectively. By integrated analysis, methylation levels at above CpG sites possessed tightly negative correlation with gene expression (Fig. 4C). Accordingly, the methylation at above CpG sites might be served as negative modulator of gene expression as well as the potential biomarkers for the resistance/susceptibility phenotype against GCRV in CIK cells.

### Functional miRNA screening as the potential biomarkers of the resistant/susceptible trait

miRNAs are proverbially recognized as the major negative players in gene expression regulation by inducing direct mRNA degradation or translational inhibition^11^. Consequently, integrated analysis of DETs and DEGs between R2 and S3 was carried out to reveal the regulatory mechanism of the resistance/susceptibility against GCRV in CIK cells at the post-transcriptional level. The results indicated that 705 genes were up-regulated by lower expressed miRNAs as well as 166 genes were impeded by overexpressed miRNAs between R2 and S3 groups (Fig. 4A). By further screening the above DETs, four representative immune-related genes, involving *LGP2*, *NLRC5*, *SOCS3B* and *TBK1 binding protein*, were indicated to be negatively modulated by mir-34-y, mir-722-y and novel-m0076-5p respectively and designated as the potential biomarkers for resistance/susceptibility against GCRV in CIK cells. Thereupon, the dual luciferase reporter assay was performed to verify the relationship between the miRNA and the corresponding predicted target. The results showed that luciferase activity of pmirGLO-WT vectors were all significantly restrained, in parallel with the luciferase activity of pmirGLO-MUT vectors without significant discrepancy or strengthened compared with the NC groups post miRNA mimics transfection, demonstrating the candidate targets were modulated by the corresponding miRNAs and identified as the potential biomarkers for the phenotypic trait in CIK cells (Fig. 4D).

## Discussion

GCRV is a double-stranded RNA (dsRNA) virus pertaining to the genus *Aquareovirus* which could cause severe hemorrhagic disease with approximately 85% mortality in fingerling and yearling *C. idella* as well as the enormous economic losses^2, 3^. Over the past decades, with the progresses of molecular and cellular biology technologies, multifarious GCRV detection techniques have been developed such as the reverse transcription loop-mediated isothermal amplification, antigenic serodiagnosis and nucleic acid sequence-based amplification combined with enzyme-linked immunosorbent assay^34–36^. And more attempts are focused on proposing the effective preventive and therapeutic strategies for hemorrhagic disease. The most direct approaches are the exploitation of vaccines and medicines, of which the viral vaccines have experienced three stages: inactivated vaccine and attenuated vaccine, recombinant subunit vaccine and DNA vaccine^37–39^. And mycophenolic acid, chestnut, and quebracho woods have been confirmed to specifically inhibit GCRV^40, 41^. Beyond that, some original antiviral strategies have been proposed to block the virus proliferation, such as RNAi, GCRV interfering particles and protease inhibitor strategies^2, 42, 43^, or in the other way to improve the antiviral immunity of organism in response to viral invasion, such as the immune adjuvant CpG oligodeoxynucleotide (ODN) method^44^. Although the above strategies provided novel avenues undeniably, grass carp hemorrhagic disease has not been controlled completely so far. Taken together, it is crucial to thoroughly revealing the regulatory mechanism of antiviral immunity in *C. idella* from the perspectives of genetic and epigenetic levels.

In the present study, transcriptome analysis elucidated that R2 possessed the stronger antioxidant activity and multiplication capacity while S3 held stronger apoptotic activity. Those pathways might be crucial to determining the discrepant resistance/susceptibility traits against GCRV (Fig. 2A,D). It was deserved to be mentioned that the most of immune-related genes were in higher expression state in S3, implying unstable state of the susceptible cells compared with the resistant ones, which could be interpreted as an error or deviation from a healthy state by the biosemiotic entropy theory^45^. That might be another crucial factor determining the resistance or susceptibility and the potential pathogenesis causing the hemorrhagic disease.

To explore the epigenetic regulatory mechanisms on the antiviral differential gene expression, we investigated the DNA methylation profiles and miRNA expression patterns in R2 and S3. It has been demonstrated gene expressions of *RIG-I-like receptors* could be negatively-regulated by the aberrant methylation post GCRV infection in our previous studies^46–48^. In accordance, our data implied that the antiviral gene expressions were extensively modulated by DNA methylation (Fig. 3A). Intriguingly, the proportion of triple nucleotides mCHH was the highest in CIK cells rather than the most common functional mCG which has been reported in zebrafish (*D. rerio*) and half-smooth tongue sole (*Cynoglossus semilaevis*) (Supplementary Fig. S6)^22, 49, 50^. In vertebrates, mutation occurred in mCG is the most frequent among all base substitutions, and the significantly reduced mCG in *C. idella* might be closely related to the increasing deamination rates which potentially raise numerous nonsynonymous substitutions and subsequent amino acid replacements for driving the evolution or adapting to varying environmental stresses^51, 52^. Hence, high mCHH methylation distribution may be a characteristic modulator in *C. idella*. By comparison with the previous researches, this study first comprehensively depicted the miRNA expression profiles in CIK cells and identified 1337 miRNAs, with 673 novel miRNAs enormously replenishing the miRNA database of *C. idella* ^30, 31^. Most importantly, 36 DE-miRNAs were identified between the resistant and susceptible groups. This might provide some hitting-points and open up a new prospect for revealing the antiviral gene expression at post-transcriptional level (Fig. 3D).

According to the integrated analysis of DNA methylation and mRNA expression patterns, a number of hyper-methylated genes were identified with lower expression level in susceptible cells, such as *CD80-like protein*, *AHCY-like 1* and *B2 bradykinin receptor-like* (Fig. 4C). *CD80* and *CD86*, the two most important cell co-stimulatory molecules, interact with *CD28* triggering the production of *interleukin-2* by activated T cells^53–55^. It has been indicated that the failure of immune surveillance mechanisms in non-inflammatory colon carcinogenesis may be linked to genomic methylation directly or indirectly affecting *CD80* expression^56^. In this study, the methylation of CGI in the 13^th^ intron was investigated increased, while the expression was decreased in S3 compared with R2, manifesting that *CD80-like* may contribute to the resistance. *AHCY* is regarded as one of the most potent methyltransferase inhibitors^57^. And it has been reported that *AHCY* deficiency may represent an ideal model disease for studying the molecular origins and biological consequences of DNA hypermethylation due to impaired cellular methylation status^58^. Consequently, it could be legitimately deduced that *AHCY-like 1* expression in S3 was restrained by hypermethylation. *B2 bradykinin receptor* could induce cell proliferation and modulate the activity of antioxidant enzymes^59–61^. Our data implied that the expression of *B2 bradykinin receptor-like* might be impeded by the hypermethylation at +692 nt, which might alter the antioxidant and multiplication capacity of S3. Beyond that, it has been demonstrated that a series of representative immune-related genes involving *LGP2*, *NLRC5*, *SOCS3B* and *TBK1 binding protein* were negatively-modulated by the oriented miRNAs (Fig. 4D).

Taken together, this study first acquired the resistant and susceptible monoclonal cells and established novel research models for pathogenesis mechanism of hemorrhagic disease. And it is the first time to combine DNA methylation, mRNA and miRNA patterns in the resistance against GCRV study. The results unravelled discrepant mRNA expression patterns between the resistance and susceptibility traits, illuminated the antiviral mechanisms against GCRV from epigenetic perspective and provided a series of potential biomarkers for resistance breeding. This study opened novel pavements for the future researches on hemorrhagic disease in *C. idella*.

## Methods

### Statement

All experiments were approved by the Animal Care Committee of Huazhong Agricultural University. The Administration of Affairs Concerning Animal Experimentation Guidelines stated approval from the Science and Technology Bureau of China. The present study does not involve in animals. CIK cells and GCRV are common cell line and virus. The methods were carried out in accordance with the approved guidelines. Approval from the Department of Wildlife Administration is not required for the experiments conducted in this paper.

### Cell culture and single cell line obtaining

CIK cells, obtained from China Center for Type Culture Collection, were cultured in Dulbecco’s modified Eagle’s medium (DMEM, Gibco, USA) supplemented with 10% fetal bovine serum (FBS, Gibco, USA) at 28 °C with 5% CO_2_. To obtain single CIK cell, CIK cell line were digested by 0.5% tyrisin, then passed through a 150 μM nylon mesh filter twice to guarantee CIK cells singly suspended in phosphate-buffered saline (PBS). 100 μL DMEM supplemented with 7% FBS, 100 U/mL of penicillin, 100 U/mL of streptomycin sulfate and 5 μL/mL insulin (Gibco, USA) were added to each well of the 96-well culture plates beforehand, the single cell was ultimately instilled in a row utilizing the BD FACSAria™ III Cell Sorter (USA). The sorted single cells were similarly maintained at 28 °C with 5% CO_2_ and sub-cultured up to being the monoclonal cells.

### Identification of the resistant and susceptible cells

All the monoclonal cells were seeded in 96-well plates at a cell density of 5 × 10^4^/well, and identified as resistant, susceptible or ambiguous by following strategies: (1) CPE analysis. Cell morphology, CPE as well as survival were photographed at 0, 6, 12, 24, 48, 72, 96 and 120 h post GCRV infection employing Leica DMI6000 B inverted deconvolution microscope (Germany) with a 10 × lens, preliminarily identifying the resistant and susceptible cells. The experiment was repeated once. (2) Cell proliferation assay. The preliminarily identified resistant and susceptible cells were further screened by detecting cell proliferation post GCRV infection using the MTT assay. CIK cells before sorting were served as the control in this test. Briefly, 20 μL of 5 mg/mL MTT was put into each well in 96-well plates at 0, 48, and 72 h post GCRV infection and incubated at 28 °C for 4 h, and then the medium was replaced by 150 μL of DMSO (Sigma, USA) and shaken at room temperature for 10 min. The absorbance (A) was measured at 570 nm. Cell proliferation was evaluated by CV which was calculated as following equation: CV = (A _48 h_ – A _0 h_) + (A _72 h_ – A _0 h_). (3) Antiviral activity assay. The resistant and susceptible monoclonal cells were eventually ascertained and reconfirmed by antiviral activity assay. At 0, 6, 12, 24, 48, 72, 96 and 120 h post GCRV infection, cells were fixed with 4% paraformaldehyde for 10 min at room temperature, orderly stained with 0.2% (wt/vol) crystal violet for 15 min, then washed with water and drained, ultimately photographed by the imaging system.

### RNA-Seq

The same number of the identified resistant and susceptible monoclonal cells were consisted of the resistant pool (R2) and the susceptible pool (S3), respectively. CIK cells before sorting were served as the control group (C1) (Supplementary Fig. S1). The integrity of above extracted RNA were confirmed using the Agilent 2100 Bioanalyzer (Agilent Technologies, USA), and estimated whether meeting the requirements of building RNA-Seq libraries by the RNA concentration (OD > 100 ng/μL), agarose gel electrophoresis (AGE) as well as RNA Integrity Number (RIN) value (RIN > 7.5). The quality controls of RNAs for building RNA-Seq and small RNA-Seq libraries were shown in Supplementary Fig. S2. mRNA was enriched by Oligo(dT) magnetic beads, and ribosomal RNA (rRNA) was removed by Ribo-Zero^TM^ Magnetic Kit (Epicentre, USA). Then the enriched mRNAs were fragmented into short pieces (200–500 nt) and reversely transcribed into cDNA with random primers. cDNA fragments were purified by QiaQuick PCR extraction kit (Qiagen, Germany), end repaired, poly (A) added, and ligated to Illumina sequencing adapters. The products were size-selected by AGE, PCR amplified, and paired-end (2 × 125 bp) sequenced using Illumina HiSeq^TM^ 2500.

### Bioinformatics analysis on RNA-Seq and qRT-PCR validation

Raw reads were filtered, removing low quality data which contain adapters, the ratio of unknown nucleotides (N) more than 10% per read and quality score under 20 (Q-value ≤ 20), to get high quality clean reads. The reads to rRNAs or tRNAs were removed by short reads alignment tool Bowtie 2^62^. Each sample was then mapped to reference genome by TopHat2^63^ (version 2.0.3.12). The reconstruction of transcripts was carried out by software Cufflinks, and transcripts were merged from multiple groups into a comprehensive set of transcripts using software Cuffmerge for further downstream differential expression analysis^64^. The expression level was normalized by FPKM (Fragments Per Kilobase of transcript per Million mapped reads) method. The edgeR package (http://www.rproject.org/) was applied to identify DEGs across groups, of which genes with a fold change ≥ 2 and a false discovery rate (FDR) < 0.05 were deemed to be the significant DEGs. DEGs were whereafter subjected to enrichment analysis of GO functions (http://www.geneontology.org/) and KEGG pathways^65^.

Total RNAs were extracted according to the manufacturer’s instruction and incubated with RNase-free DNase I (Roche, USA) to eliminate contaminated genomic DNA, subsequently being reversely transcribed into cDNA using random hexamer primers and M-MLV Reverse Transcriptase (Promega, USA). A portion of gene expression were detected by qRT-PCR, according to previously described procedure^66^, not merely verifying the reliability of sequencing data, but also sustaining the legitimate inferences based on the above analysis. Primer sequences for qRT-PCR are listed in the Supplementary Table S1.

### MeDIP-Seq and small RNA-Seq

For ascertaining the epigenetic mechanism of resistance traits against GCRV in CIK cells, DNA methylation and small RNA libraries were built in C1, R2 and S3 groups. The DNA of each group, extracted by the classical phenol and chloroform method, was sonicated into 100–500 bp fragments. Then libraries were constructed following the Illumina Paired-End protocol consisting of end repair, < A > base addition, sequencing adaptor concatenation and double-stranded DNA (dsDNA) degeneration. Adaptor-ligated DNA was immunoprecipitated by 5′-methylcytidine antibody and validated by quantitative PCR. The enriched methylated DNA were amplified, and the fragments of 220–320 bp in size were gel-excised and quality was controlled by Agilent BioAnalyzer analysis, ultimately paired-end (2 × 125 bp) sequenced using Illumina HiSeq^TM^ 2500 to build the methylation libraries. Raw reads were filtered, then implemented the alignment analysis to obtain the uniquely mapped reads. DMRs between groups, with a fold change of coverage ≥ 2 and FDR < 0.01, were carried out enrichment analysis of GO functions and KEGG pathways, as well as distribution analyses.

RNA molecules in C1, R2 and S3 groups were enriched in a size range of 18–30 nt by polyacrylamide gel electrophoresis (PAGE), respectively. The 3′ adapter and 5′ adapter were then ligated. RNAs were reversely transcribed and amplified by PCR. Ultimately, the 140–160 bp PCR products were enriched to generate a cDNA library and sequenced using Illumina HiSeq^TM^ 2500. All tags were filtered to get clean data which were aligned with small RNAs in GenBank database (Release 209.0) and Rfam database (11.0) to identify and remove rRNA, scRNA, snoRNA, snRNA and tRNA. Subsequently, they were aligned with reference genome to remove fragments mapped to exons, introns and repeat sequences. The known and exist miRNAs were identified in virtue of the miRBase database (Release 21), and the novel miRNA candidates were predicted by software Mireap_v0.2 according to their genome positions and hairpin structures. The miRNA expression level was calculated and normalized to transcripts per million (TPM). DE-miRNAs were defined based on the general principles (fold change ≥ 2, *P* < 0.05). The candidate target genes coupled with miRNAs were predicted by three softwares RNAhybrid (v2.1.2), Miranda (v3.3a) and TargetScan (Version: 7.0), and the intersection of these results would provide more credibly predicted miRNA target genes. The identified DETs were then carried out into GO functions and KEGG pathways, as well as distribution analyses.

### 5-mC in total DNA and BSP

Global DNA methylation status in C1, R2 and S3 groups were detected using the MethylFlash^TM^ Methylated Quantification DNA Kit (Epigentek, USA) according to the manufacturer’s instructions. The input DNA amount was 100 ng for optimal quantification. The 5-mC is detected using capture and detection antibodies and then quantified colorimetrically by reading the absorbance in the Infinite^®^ 200 Pro NanoQuant (Tecan, Switzerland). To quantify the absolute amount of methylated DNA, the multiple concentrations of ME4 were served as the positive controls and a standard curve plot, and ME3 was served as the negative control. The amount and percentage of methylated DNA in total DNA were calculated using the following formulas, respectively: 5-mC (ng) = (Sample OD – ME3 OD)/(Slope × 2); 5-mC % = [5-mC (ng)/100 ng] × 100%. The slope (OD/ng) of the standard curve was obtained using linear regression; 2 is a factor to normalize 5-mC in the positive control to 100%; 100 ng is the amount of DNA per sample.

The significantly discrepant methylation statuses in the representative genes between R2 and S3 groups excavated from the MeDIP-Seq were verified by BSP. 1 μg of DNA was bisulfite-modified using the EZ DNA Methylation-Gold^™^ Kit (Zymo, USA) according to the manufacturer’s instruction. The specific methylation primers for BSP were designed by the Methprimer, verified in virtue of Methyl Primer Express v1.0 (Applied Biosystems, USA) and Primer Premier 5 (PREMIER Biosoft, USA) and shown in the Supplementary Table S2. The DNA fragments of BSP assays was cloned into the pMD18-T (TaKaRa, Japan). Twelve positive clones from each group were randomly selected for sequencing. The results were analyzed by QUMA software (http://quma.cdb.riken.jp/).

### qRT-PCR analysis of miRNA

1 μg of total RNA was performed poly(A) tailing and cDNA synthesis using the mi*DETECT* A Track^TM^ miRNA qRT-PCR Starter Kit (Ribobio, China) according to the manufacturer’s instructions. miRNA was reverse transcribed by the Uni-RT primer which involves the universal reverse primer sequence for qRT-PCR. The forward primer sequences were listed in the Supplementary Table S3. miRNA expression and data analysis were similar to those described previously^66^. Expression levels of target miRNAs were normalized to U6 small nuclear RNA (snRNA).

### 3′UTR dual luciferase reporter assay

The template cDNA were obtained by reverse transcription with the 3′ RACE universal adaptor primer. The primers for vector construction were listed in the Supplementary Table S4. The PCR products were cloned into the downstream of Firefly luciferase’s open reading frame (ORF) in pmirGLO Dual-Luciferase miRNA Target Expression Vector (Promega, USA) which also contains an independently expressed Renilla luciferase gene as transfection control. Point mutation in the seed region within the 3′UTR of target gene was generated using overlap-extension PCR. All constructs were confirmed by sequencing. Fathead minnow (FHM) cells were plated into 24-well plates beforehand, co-transfected with 0.2 μg of 3′UTR dual luciferase reporter vector and miRNA mimic or negative control mimic (GenePharma, China) by FuGENE 6 (Promega, USA). Luciferase activity was measured using the DualGlo Luciferase Assay System (Promega, USA). Firefly luciferase activity was normalized by the corresponding Renilla luciferase activity and plotted as a percentage of the control. Experiments were performed in triplicate wells of a 24-well plate and repeated in quadruplicate.

### Statistical analysis

All of the results, except for the BSP statistical analysis, were assessed via Analysis of Variance (ANOVA) followed by Independent-Sample T Test. The discrepancies of methylation levels between R2 and S3 groups at CpG loci in the CGIs were analyzed by the 2 × 2 Chi-square (χ^2^) test with SPSS 19.0 software (SPSS Inc.). *P*-values less than 0.05 and 0.01 were considered statistically significant and extremely significant difference, respectively.

### Data availability statement

The data of RNA-Seq, MeDIP-Seq and small RNA-Seq in this study have been submitted to Gene Expression Omnibus (GEO, http://www.ncbi.nlm.nih.gov/geo/) under accession number GSE87414.

## Electronic supplementary material

Supplementary information

Dataset 1

Dataset 2
