# Supplementary material for: The destiny of the resistance/susceptibility against GCRV is controlled by epigenetic mechanisms in CIK cells
Source: Sci Rep. 2017 Jul 3;7:4551. doi: 10.1038/s41598-017-03990-5 (PMC5495752; doi:10.1038/s41598-017-03990-5)
Supplement: Supplementary file 1 — Supplementary information [file 41598_2017_3990_MOESM1_ESM.pdf]

The destiny of the resistance/susceptibility against GCRV is controlled by epigenetic mechanisms in CIK cells

Xueying Shang[1], Chunrong Yang[2], Quanyuan Wan[3,4], Youliang Rao[3,4] & Jianguo Su[1,3,4*]

[1]*College of Animal Science and Technology, Northwest A&F University, Yangling 712100, China*

[2]*College of Veterinary Medicine, Huazhong Agricultural University, Wuhan 430070, China*

[3]*College of Fisheries, Huazhong Agricultural University, Wuhan 430070, China*

[4]*Hubei Provincial Engineering Laboratory for Pond Aquaculture, Wuhan 430070, China*

[*]Corresponding author, Tel/Fax: 86-27-87282227; E-mail address: sujianguo@mail.hzau.edu.cn

Keywords: Grass carp (*Ctenopharyngodon idella*); Integrated omics; RNA-Seq; DNA methylation; microRNA; Grass carp reovirus

Supplementary information

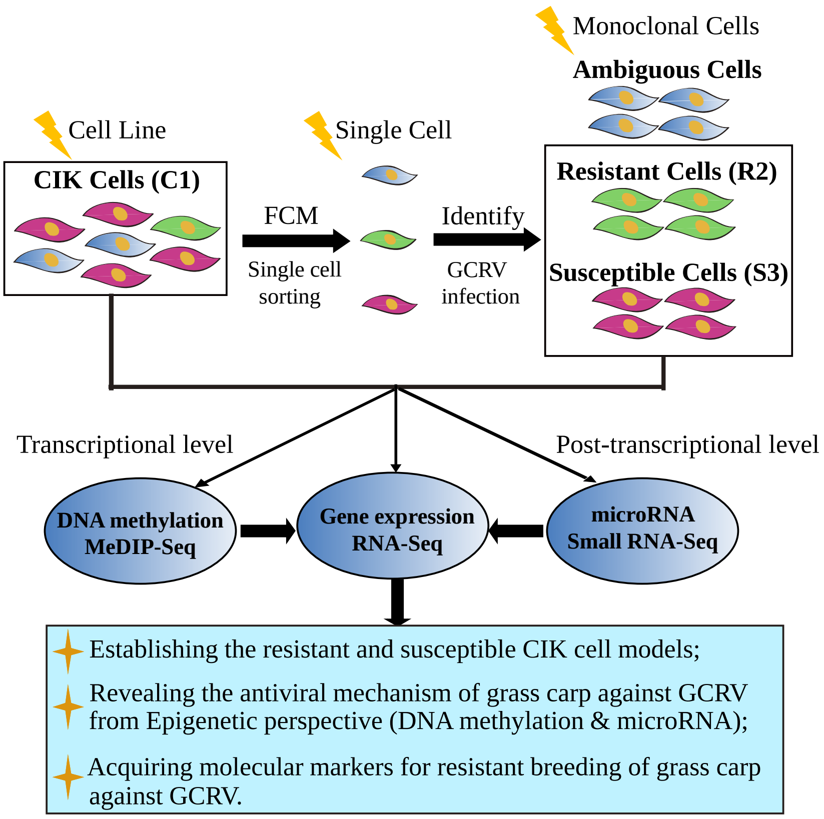

Supplementary Fig. S1. Scheme depicting the strategy designed in this study. Genomic DNA methylation patterns, mRNA and miRNA expression datasets were integrated, using resistant and susceptible monoclonal cells (R2 and S3) as experimental groups and CIK cell line (C1) as control. The upper area shows the method for establishing the resistant and susceptible monoclonal cell models. The cell samples in boxes were prepared for high-throughput sequencing. The middle region displays the research strategies in this study which revealed regulatory mechanism of the resistance/susceptibility against GCRV from epigenetics perspective, involving DNA methylation at transcriptional level (left) and miRNAs at post-transcriptional level (right). The establishment of cell models and integrated analysis of sequencing data are described in detail in the results section. The lower part expounds the purpose and significance of this study.

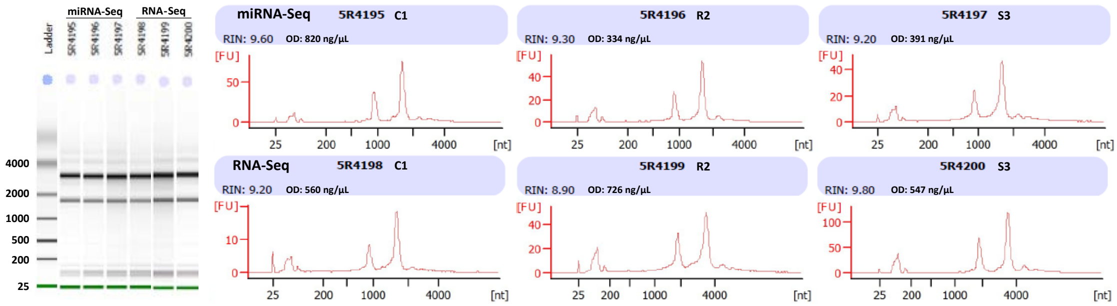

Supplementary Fig. S2. RNA quality control for building libraries. The left graph exhibits the results by agarose gel electrophoresis (AGE). The right one shows the integrity of the extracted RNA using the Agilent 2100 Bioanalyzer.

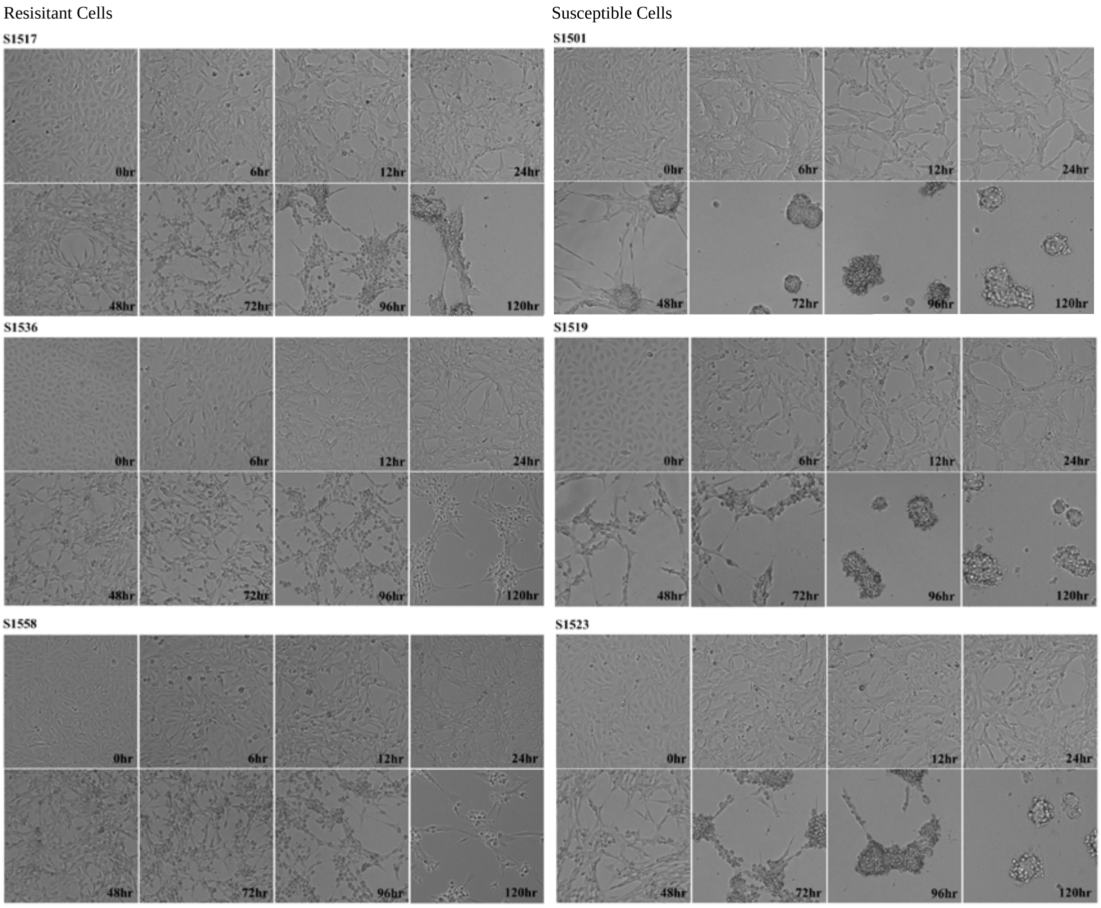

Supplementary Fig. S3. The morphology and CPE of the resistant (S1517, S1536, S1558) and susceptible (S1501, S1519, S1523) cells at 0, 6, 12, 24, 48, 72, 96 and 120 h post GCRV infection.

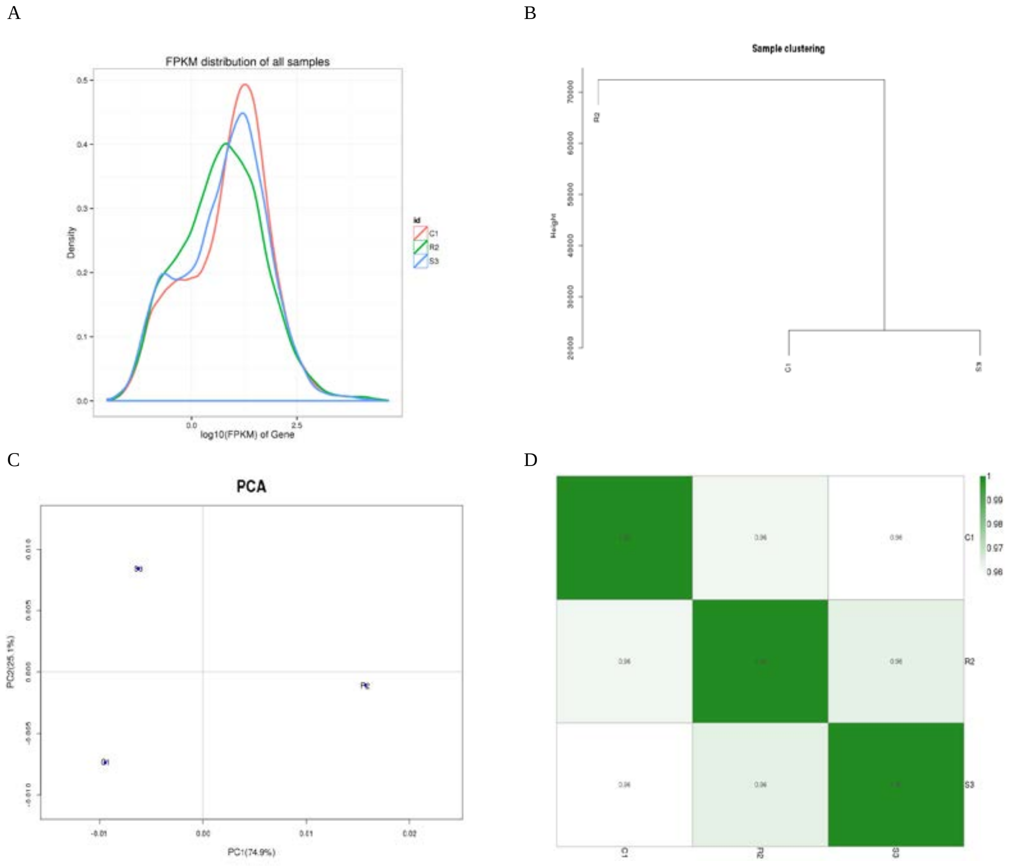

Supplementary Fig. S4. mRNA expression patterns among samples. (A) Abundance distribution of mRNA expression. (B) Clustering analysis. (C) Principal component analysis (PCA). (D) Correlation heat map.

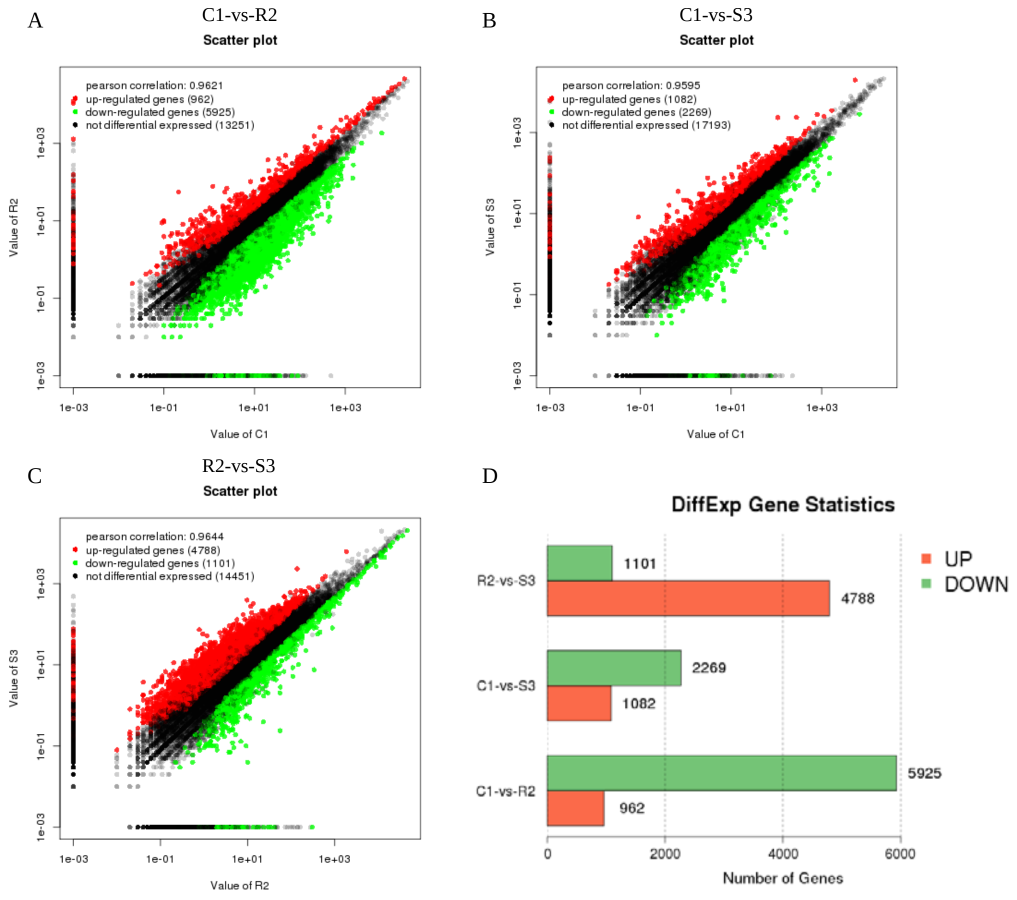

Supplementary Fig. S5. Statistics of DEGs among samples. The red spots and columns represent the gene expression higher in the latter than those in the former, the green ones signify conversely.

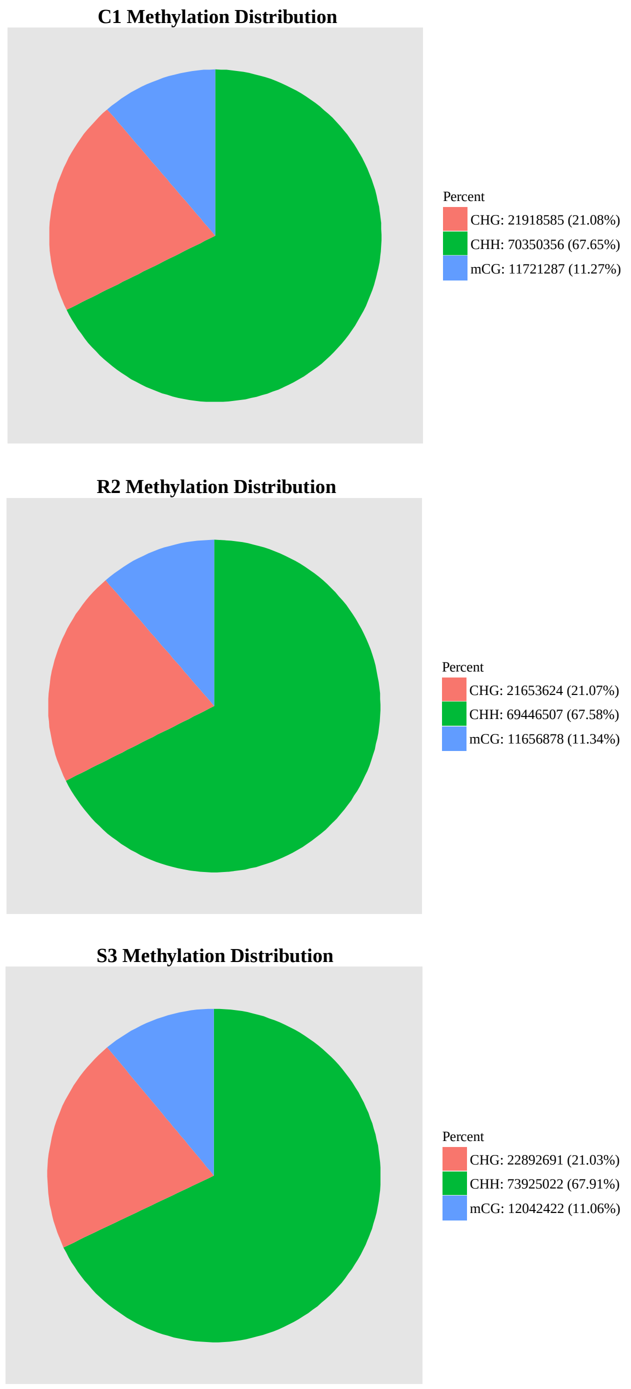

Supplementary Fig. S6. Distribution of mC in C1, R2 and S3 (mCG, mCHG and mCHH, where H = C, T, or A).

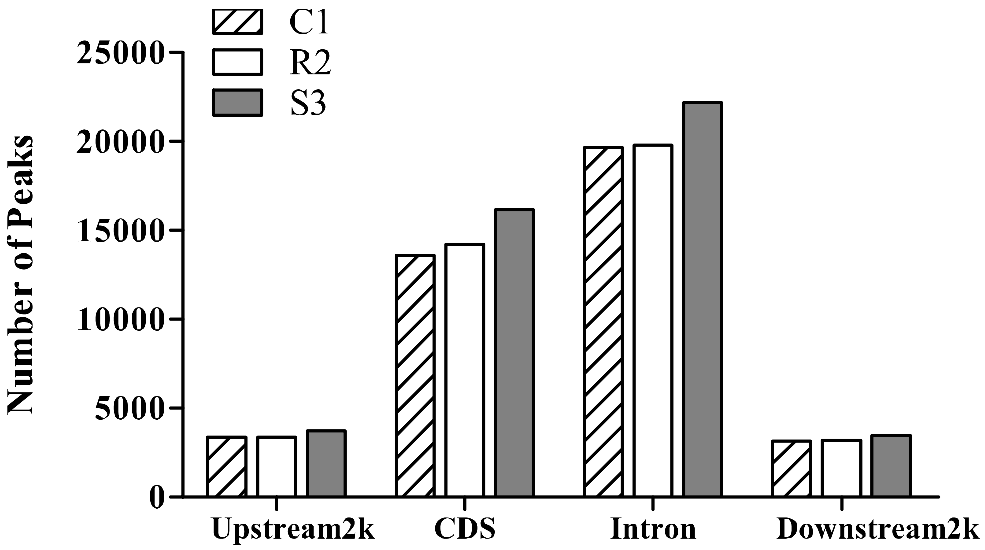

Supplementary Fig. S7. The statistics of peaks located in the different functional elements, including upstream2k, CDS, intron and downstream2k.

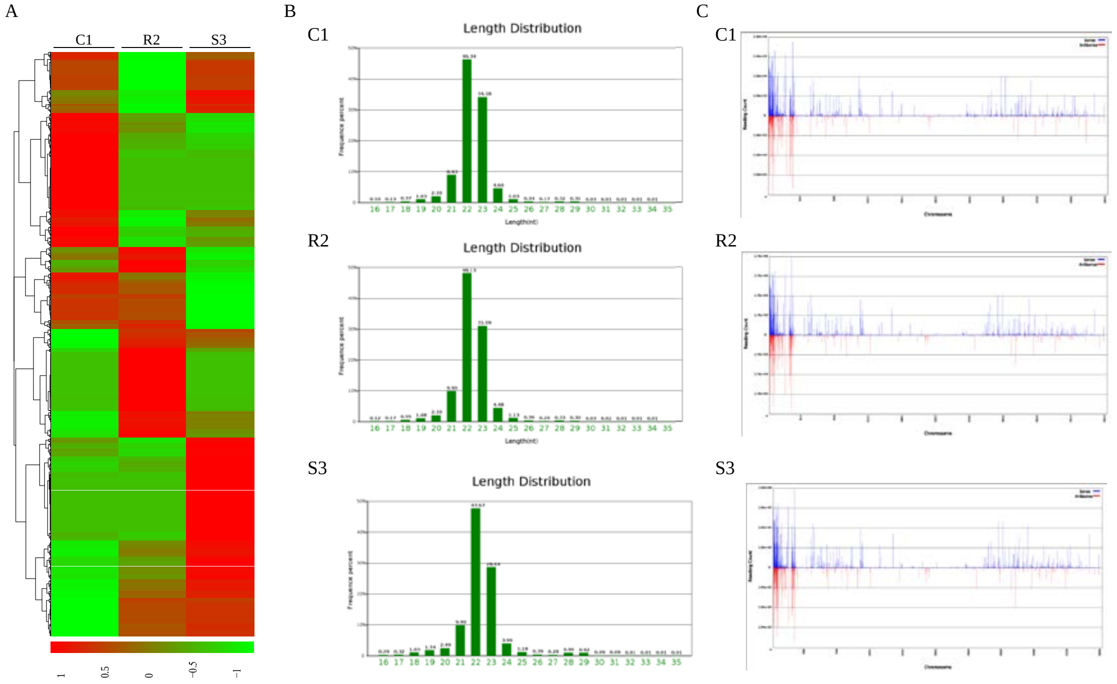

Supplementary Fig. S8. The expression profiling (A), length statistics (B) and chromosomes distribution of miRNAs in C1, R2 and S3 (C).

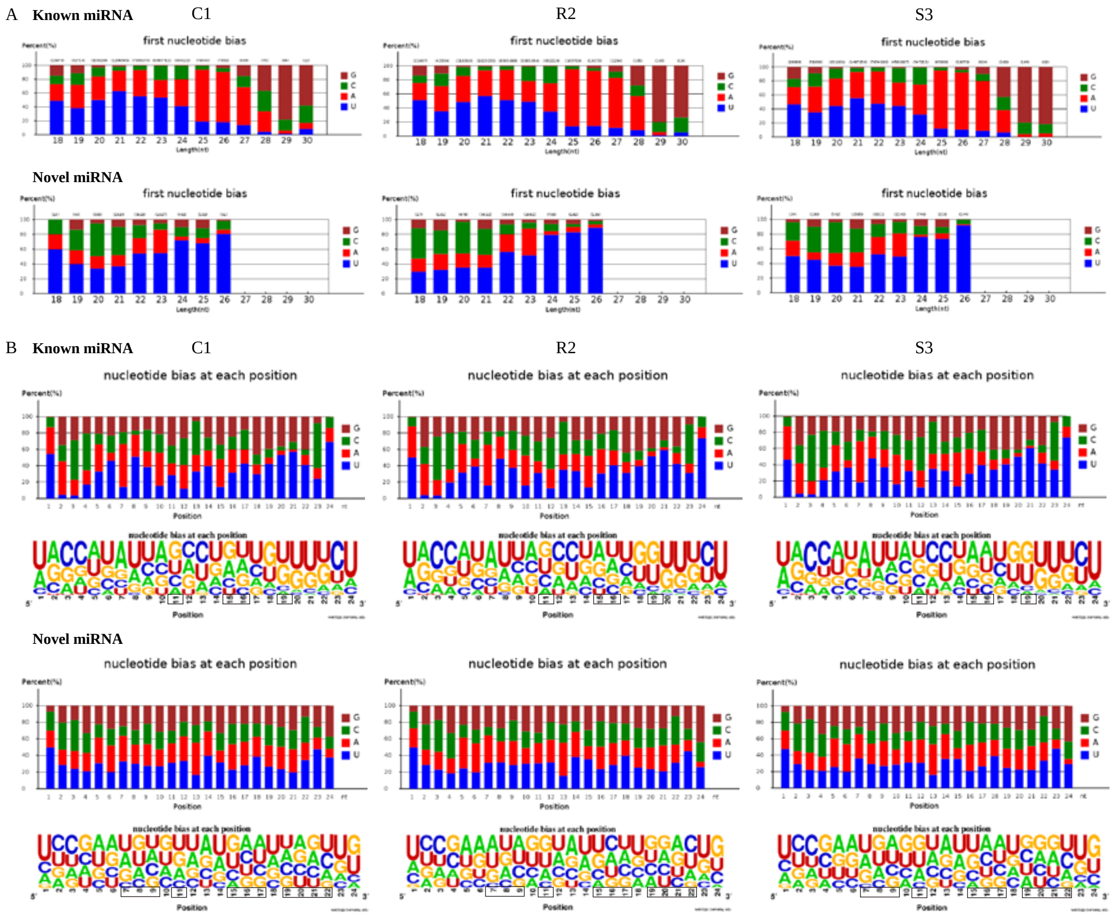

Supplementary Fig. S9. The nucleotide bias analysis of known and novel miRNAs. (A) The first nucleotide bias. (B) The nucleotide bias at each position. The positions labeled by boxes are the discrepant loci among three groups.

Supplementary Table S1

Sequences and amplicon lengthes of primers designed for mRNA qRT-PCR in this study.

Primer name	Sequence (5’-3’)	Amplicon length (bp)
S-adenosylhomocysteine hydrolase-like 1		
CF01	CAGACTGTGGCGGGGATG	113
CR02	GTCGCTGTTCTTCGTGGCT	
TNF receptor superfamily member 4-like		
CF03	GTATAAGAGGAACTGTACGGCGAC	147
CR04	TGTGGTGTTTTAGTAGCAGAAGGAG	
CD80-like protein		
CF05	AGACTTTTGAATCTGAAGAGCCTG	115
CR06	ATTCCTAAATCATCAACAGTGACAGTAT	
B2 bradykinin receptor-like		
CF07	TTGAATGGCGATTTGGGTC	162
CR08	TGGCGTATCGTGGTCGTCT	
TNF ligand superfamily, member 10 like 3		
CF09	TCCGAAGGCAACTCCGAAT	126
CR10	GATAACAACAACCGTCGTCACC	
methyltransferase-like protein 12		
CF11	AACCCATCCTCTGCTCTTGC	169
CR12	CCTCTAAACATTGCCTCACTATCTG	
suppressor of cytokine signaling 4		
CF13	ACCACCATCATCACCAGTCC	194
CR14	GGGTTCGGTTTCAATGCTTA	
polymeric immunoglobulin receptor-like		
CF15	GACCAGACATCTACAGCATCACC	206
CR16	GTGGGTTCAAATATCACATCCTTAG	
deltex1		
CF17	CAACAACGGCAACAAAGACG	122
CR18	CAGGAAGGGAGTGAGGAATGAC	
RNA helicase LGP2		
CF19	GGACACCTCAACCTCCTCATC	164
CR20	TCAGCCACCACCGAGTAGAC	
NLRC3-like		
CF21	TAAGGGCTTTTCAAGGACTGC	94
CR22	CTTCACAGCCTCTTCTGGTCC	
NLRC5-like		
CF23	CAAATCCTGTTGGCGTTCCT	139
CR24	TGAAGACTATGGTGCTTTCCTCC	
NLR family member X1		
CF73	AACAAGGCACGAGGACTAAATG	141
CR74	CTCACAGGAGAAGGGCAACAC	

TBK1-binding protein 1-like		
CF27	CAGAGGATGAGGAGGAGTGGTC	131
CR28	GTTGGGACGGGAAGGAATG	
interleukin 3 regulated, member 5		
CF29	AGAAGCCTCCACTGACGATG	155
CR30	GCAGAGCAGTGCCTTTCATT	
interleukin-17 receptor D		
CF31	AGTCTCGCTTCAAGATACACCCT	133
CR32	GGAGTTTACAGTATCCGCTTTCAG	
IRF2-binding protein 1		
CF33	CTCCGAAAGTGTAAGGAACCG	199
CR34	GCACGGGTACTCGACGAAC	
TNF α-induced protein 8-like protein 1		
CF37	TGGTCAAGTTGGGCGTCCT	173
CR38	GCGGCAGTCGTTGAGTAAGTT	
immunoglobulin superfamily member 21-like		
CF41	TCAGCAACACGCACATTCC	112
CR42	GTGACCTCGCAGCTAAACAAG	
macrophage-stimulating protein receptor		
CF43	GTCTACAACGTGGTTCAAGCG	125
CR44	ACACCCAAGTCATCAGTTACAGC	
MHC class I protein		
CF45	TCTCCAGATGTTCGTGTCTCTG	127
CR46	GGATGTTTCTGTACAATCTAATGTTC	
suppressor of cytokine signaling 3b		
CF47	GGCTTCTACTGGAGCACCGT	181
CR48	GCAGGAAGAAGGAGCAGGAAT	
immediate early response gene 5		
CF51	GGGGAATCACATCAAGCACC	138
CR52	CTCGCCTTCCTCGTCCTCT	
Id1		
CF53	GTTGGAGGCGAAGATGTTGTC	161
CR54	TTGTTGGTCGGTAGCGTGG	
cyclin-dependent kinase inhibitor 1B		
CF55	CTCCAATCTTCATTTGTGCTCCT	114
CR56	CGAGGTGTTTCGGGCGT	
tumor protein p53-inducible nuclear protein 1		
CF57	ATTCGGCGGCAGACACC	186
CR58	CGTACACGGACATGCTGGG	
BCL2/adenovirus E1B interacting protein 3-like a		
CF59	GGTGGAGCTGGAGTTGAACG	170
CR60	TAGAGGAGGAGGAAGGCACG	
glutathione peroxidase 3 (plasma) precursor		
CF63	CAGACGGAAGACCAGTAATGAGG	84

CR64	GCTGGCGGAAGTATTTCAAGAT	
succinate dehydrogenase complex		
CF65	CACTTGCTGCGTTGGTTTTAC	141
CR66	GGATGCCGTTGAAGGTGTG	
acetyl-CoA carboxylase 2 isoform X2		
CF77	CGGTACGGTAGTCGTCTGTGG	151
CR78	CCTCCTTGTATAGGCTGATGTCC	
lipoprotein lipase		
CF71	TGAACCAGGAGCAACAAAGC	144
CR72	TTCTGCTGCTCCTGCGTG	
EF1α		
EF125	CGCCAGTGTTGCCTTCGT	99
ER126	CGCTCAATCTTCCATCCCTT	

Supplementary Table S2

Sequences and amplicon lengthes of primers designed for BSP in this study.

Primer name	Sequence (5'-3')	Amplicon length (bp)
S-adenosylhomocysteine hydrolase-like 1		
MCF87	GTTGTGATTAGGGAGTATTTGGAT	268
MCR88	ACCCTACTACTCACGAAACATCTAC	
MCF89	GATGTTTCGTGAGTAGTAGGGTT	263
MCR90	ACCCTCTCCCAAATAAACTCT	
CD80-like protein		
MCF91	AATAAGGCGATATGTTAGTGG	238
MCR92	TTATAACTAAAACTCTCTATCTAACTACTT	
MCF93	AAAGTAGTTAGATAGAGAGTTTTAGTTAT	290
MCR94	TTCCTTTATACAACTAAACATACATAC	
B2 bradykinin receptor-like		
MCF103	AATTGGTCGGAGTTGTTGAG	326
MCR112	AATCAATCATTTATTCACACAATTAC	

Supplementary Table S3

Primers sequences designed for miRNA qRT-PCR in this study.

miRNA	Primer name	Sequence (5’-3’)
mir-34-y	M34-yF6A	GAATCAGCAAGAATACTGCCGTTA
novel-m0076-5p	M0076-5pF7	GTTGCTTTAGTGCTCTTGCTTGTA
mir-135-x	M135-xF8	GGCTGTTATGGCTTTCTATTCCT
mir-722-y	M722-yF9A	GCCTTTTTTCCAGTATCGTTTCA
mir-191-x	M191-xF10	CAACGGAATCCCAAAAGCAG
mir-730-x	M730xF11	GGCTCCTGATTGTGAATGCTG
mir-460-y	M460-yF12	GCTCAGGTTCACACCGCATA
mir-730-y	M730-yF13	GCTACAAGTTCACACCGCCTG
U6 snRNA	U6F16	CGCTTCGGCAGCACATATAC
	U6R17	TTCACGAATTTGCGTGTCATC

Supplementary Table S4

Sequences, applications and amplicon lengthes of primers for luciferase reporter assay in this study.

Primer name	Sequence (5'-3')	Application and amplicon length (bp)
RNA helicase LGP2		
LLF1	**ACTG**gctagcAGCTGTCACTGTGCCAGAATT	pLGP2-WT
LLR2	**ACTG**tctagaGCAACCGTAAGGACGAAAAC	823
LLF11	AGCTGTCACTGTGCCAGAATTTCTCTCAGAT TTGTGAATATCTCTATACCAGTGGCAG	pLGP2-MUT (novel-m0076-5p)
LLR12	CTGCCACTGGTATAGAGATATTCACAAATCT GAGAGAAATTCTGGCACAGTGACAGCT	58
LLF13	TGAATATCTCTATACCAGTGGCAG	
LLR2	**ACTG**tctagaGCAACCGTAAGGACGAAAAC	789
LLF1	**ACTG**gctagcAGCTGTCACTGTGCCAGAATT	pLGP2-MUT (mir-34-y)
LLR14	GATGTAAAAAGTCGATAATCTGGATAG	147
LLF15	CTATCCAGATTATCGACTTTTTACATC	
LLR2	**ACTG**tctagaGCAACCGTAAGGACGAAAAC	703
NLRC5		
LNF3	**ACTG**gctagcAAATAACCACGGCAGGAGC	pNLRC5-WT
LNR4	**ACTG**tctagaGGTTGTGCGTTTACCAGTGAC	770
LNF3	**ACTG**gctagcAAATAACCACGGCAGGAGC	pNLRC5-MUT (mir-34-y)
LNR16	TCAGTGTAAGTCGAAAGAGCATAAC	537
LNF17	GTTATGCTCTTTCGACTTACACTG	
LNR4	**ACTG**tctagaGGTTGTGCGTTTACCAGTGAC	258
TBK1-binding protein 1-like		
LTF9	**ACTG**gctagcAGACAGAATGTAGGCGATGC	pTBK1-BP-WT
LTR10	**ACTG**tctagaCTTTCCCATTTTAGTTCCTCTG	1217
LTF9	**ACTG**gctagcAGACAGAATGTAGGCGATGC	pTBK1-BP-MUT (mir-722-y)
LTR18	GTGTTACTTAAAACCCTTAGTGTTAGT	432
LTF19	ACTAACACTAAGGGTTTTAAGTAACAC	
LTR10	**ACTG**tctagaCTTTCCCATTTTAGTTCCTCTG	812
suppressor of cytokine signaling 3b		
LSF22	**ACTG**gctagcCAAAGACGGACTGCTGAAGAT	pSOCS3B-WT
LSR8	**ACTG**tctagaGCACGGTCCTTCATGTAAAAC	607
LSF22	**ACTG**gctagcCAAAGACGGACTGCTGAAGAT	pSOCS3B-MUT (mir-34-y)
LSR20	TATTGAATCAAGTCGAATGTTCAGT	508
LSF21	ACTGAACATTCGACTTGATTCAAT	
LSR8	**ACTG**tctagaGCACGGTCCTTCATGTAAAAC	124

Note: "ACTG" in bold represents protective bases.

Supplementary Table S5

The mapping results with *C. idella* genome.

	C1	R2	S3
**Clean base (nt)**	5,930,031,750	6,341,051,750	6,234,584,000
**Clean reads**	47,163,276	50,148,298	49,509,734
**Mapped reads**	39,778,122	42,654,140	42,950,397
**Mapping ratio**	84.34%	85.06%	86.75%

Supplementary Table S6

The mapping results with reference genome of the MeDIP-Seq.

	C1	R2	S3
**Total base (nt)**	5,564,137,750	5,486,812,500	6,672,529,750
**Total reads**	44,513,102	43,894,500	53,380,238
**Mapped reads**	37,436,470	37,565,439	45,846,648
**Mapping ratio**	84.10%	85.58%	85.89%

Supplementary Table S7

The information of peaks from the MeDIP-Seq.

	**C1**	**R2**	**S3**
**Peak number**	57,463	57,306	63,571
**Average length (nt)**	1294.11	1368.73	1376.30
**Coverage ratio**	8.26%	8.71%	9.72%

Supplementary Table S8

Methylation analysis between R2 and S3 cells at CpG loci in *CD80-like protein*.

Locus	Methylation status	Resistant R2 cells NO (%)	Susceptible S3 cells NO (%)	χ^2-value	*P*-value
**+18 nt**	MC	12 (100.0)	12 (100.0)	N	N
	UMT	0 (0.0)	0 (0.0)		
**+192 nt**	MC	9 (75.0)	12 (100.0)	3.429	0.064
	UMT	3 (25.0)	0 (0.0)		
**+196 nt**	MC	10 (83.3)	12 (100.0)	2.182	0.140
	UMT	2 (16.7)	0 (0.0)		
**+206 nt**	MC	10 (83.3)	12 (100.0)	2.182	0.140
	UMT	2 (16.7)	0 (0.0)		
**+290 nt**	MC	12 (100.0)	12 (100.0)	N	N
	UMT	0 (0.0)	0 (0.0)		
**+351 nt**	MC	4 (33.3)	6 (50.0)	0.686	0.408
	UMT	8 (66.7)	6 (50.0)		
**+358 nt**	MC	5 (41.7)	9 (75.0)	2.743	0.098
	UMT	7 (58.3)	3 (25.0)		
**+367 nt**	MC	6 (50.0)	10 (83.3)	3.000	0.083
	UMT	6 (50.0)	2 (16.7)		
**+381 nt**	MC	2 (16.7)	6 (50.0)	3.000	0.083
	UMT	10 (83.3)	6 (50.0)		
**+397 nt**	MC	0 (0.0)	1 (8.3)	1.043	0.307
	UMT	12 (100.0)	11 (91.7)		
**Total CpG-loci**	MC	70 (58.3)	92 (76.7)	9.193	0.002**
	UMT	50 (41.7)	28 (23.3)		

Note: MC and UMT stand for methylation and unmethylation, respectively.

Supplementary Table S9

Methylation analysis between R2 and S3 cells at CpG loci in *S-adenosylhomocysteine hydrolase-like 1*.

Locus	Methylation status	Resistant R2 cells NO (%)	Susceptible S3 cells NO (%)	χ^2-value	*P*-value
**+13 nt**	MC	8 (66.7)	12 (100.0)	4.800	0.028*
	UMT	4 (33.3)	0 (0.0)		
**+28 nt**	MC	7 (58.3)	12 (100.0)	6.316	0.012*
	UMT	5 (41.7)	0 (0.0)		
**+38 nt**	MC	7 (58.3)	12 (100.0)	6.316	0.012*
	UMT	5 (41.7)	0 (0.0)		
**+100 nt**	MC	5 (41.7)	8 (66.7)	1.510	0.219
	UMT	7 (58.3)	4 (33.3)		
**+149 nt**	MC	12 (100.0)	12 (100.0)	N	N
	UMT	0 (0.0)	0 (0.0)		
**+170 nt**	MC	12 (100.0)	12 (100.0)	N	N
	UMT	0 (0.0)	0 (0.0)		
**+212 nt**	MC	12 (100.0)	11 (91.7)	1.043	0.307
	UMT	0 (0.0)	1 (8.3)		
**+223 nt**	MC	12 (100.0)	11 (91.7)	1.043	0.307
	UMT	0 (0.0)	1 (8.3)		
**+241 nt**	MC	8 (66.7)	7 (58.3)	0.178	0.673
	UMT	4 (33.3)	5 (41.7)		
**+337 nt**	MC	9 (75.0)	9 (75.0)	0.000	1.000
	UMT	3 (25.0)	3 (25.0)		
**+389 nt**	MC	9 (75.0)	4 (33.3)	4.196	0.041*
	UMT	3 (25.0)	8 (66.7)		
**+397 nt**	MC	12 (100.0)	9 (75.0)	3.429	0.064
	UMT	0 (0.0)	3 (25.0)		
**Total CpG-loci**	MC	113 (78.5)	119 (82.6)	0.798	0.372
	UMT	31 (21.5)	25 (17.4)		

Note: MC and UMT stand for methylation and unmethylation, respectively.

Supplementary Table S10

Methylation analysis between R2 and S3 cells at CpG loci in *B2 bradykinin receptor-like*.

Locus	Methylation status	Resistant R2 cells NO (%)	Susceptible S3 cells NO (%)	χ^2-value	*P*-value
**+644 nt**	MC	12 (100.0)	12 (100.0)	N	N
	UMT	0 (0.0)	0 (0.0)		
**+692 nt**	MC	7 (58.3)	12 (100.0)	6.316	0.012*
	UMT	5 (41.7)	0 (0.0)		
**+725 nt**	MC	11 (91.7)	12 (100.0)	1.043	0.307
	UMT	1 (8.3)	0 (0.0)		
**+730 nt**	MC	12 (100.0)	12 (100.0)	N	N
	UMT	0 (0.0)	0 (0.0)		
**+744 nt**	MC	12 (100.0)	12 (100.0)	N	N
	UMT	0 (0.0)	0 (0.0)		
**+752 nt**	MC	12 (100.0)	12 (100.0)	N	N
	UMT	0 (0.0)	0 (0.0)		
**+754 nt**	MC	12 (100.0)	12 (100.0)	N	N
	UMT	0 (0.0)	0 (0.0)		
**+778 nt**	MC	11 (91.7)	12 (100.0)	1.043	0.307
	UMT	1 (8.3)	0 (0.0)		
**+807 nt**	MC	11 (91.7)	12 (100.0)	1.043	0.307
	UMT	1 (8.3)	0 (0.0)		
**+809 nt**	MC	11 (91.7)	11 (91.7)	0.000	1.000
	UMT	1 (8.3)	1 (8.3)		
**+817 nt**	MC	12 (100.0)	12 (100.0)	N	N
	UMT	0 (0.0)	0 (0.0)		
**+884 nt**	MC	10 (83.3)	9 (75.0)	0.253	0.615
	UMT	2 (16.7)	3 (25.0)		
**Total CpG-loci**	MC	133 (92.4)	140 (97.2)	3.446	0.063
	UMT	11 (7.6)	4 (2.8)		

Note: MC and UMT stand for methylation and unmethylation, respectively.

Supplementary Table S11. Information of sequences for integrated analyses.

(Excel file)

Supplementary Table S12. The potential molecules involved in the regulation on cell proliferation between the resistant and susceptible traits.

(Excel file)
